# Cyanidin-3-O-Glucoside Protects Against Cognitive Impairment in D-Galactose-Induced Aging Mice by Regulating Nrf2 and NF-κB Pathways

**DOI:** 10.3390/nu18060992

**Published:** 2026-03-20

**Authors:** Dan Sun, Yishan Bao, Qian Fan, Liang Zhao, Zhifang Fu, Hong Li, Lei Zhao, Hongmei Jiao

**Affiliations:** 1 Department of Geriatrics, Peking University First Hospital, Beijing 100034, China; 13581525356@163.com (D.S.); fuzhifang2004@126.com (Z.F.); lih7510@163.com (H.L.); 2 Key Laboratory of Geriatric Nutrition and Health, Ministry of Education, Beijing Technology and Business University, Beijing 100048, China; 13589757621@163.com (Y.B.); fanqian@st.btbu.edu.cn (Q.F.); liangzhao@btbu.edu.cn (L.Z.); 3 National Center of Technology Innovation for Dairy, Hohhot 010110, China

**Keywords:** cyanidin-3-O-glucoside, aging, cognitive impairment, oxidative stress, inflammation, Nrf2/Keap1, NF-κB

## Abstract

Background/Objectives: This study aimed to investigate the protective effects and underlying molecular mechanisms of cyanidin-3-O-glucoside (C3G) against cognitive impairment in aging mice induced by D-galactose (D-gal). Methods: Spatial learning and memory, hippocampal histopathology, oxidative stress and inflammatory markers, as well as underlying regulatory pathways, were assessed in C3G-treated D-galactose-induced aging mice via Morris water maze, H&E staining, biochemical assays, qRT-PCR and Western blot. Results: Results showed C3G improved cognitive function by reducing escape latency and increasing target quadrant time along with platform crossings, while also alleviating hippocampal damage. It dose-dependently enhanced total antioxidant capacity and activities of key antioxidant enzymes (GSH-Px and SOD), reduced malondialdehyde, and inhibited pro-inflammatory cytokines (TNF-α, IL-1β and IL-6). At the molecular level, C3G treatment was associated with changes in the Nrf2 and NF-κB pathways at mRNA and protein levels. It enhanced Nrf2 expression and reduced Keap1 expression, accompanied by upregulated mRNA levels of *Nqo1* and *Hmox1.* Meanwhile, C3G decreased IKKβ and p65 protein expression and downregulated mRNA levels of *Ikbkb*, *Nfkb1*, and *RelA*. The combined contribution of these pathways in reducing ROS and inflammation may constitute the molecular basis underlying the neuroprotective effects of C3G. Conclusions: C3G alleviates cognitive dysfunction and brain damage in D-gal-induced aging mice, with effects associated with modulation of Nrf2 and NF-κB pathways. These findings offer preliminary insights for its dietary application in brain aging intervention.

## 1. Introduction

Aging is a complex biological process characterized by the progressive decline in physiological functions and increased susceptibility to age-related diseases. The brain is particularly vulnerable to oxidative stress and inflammatory damage, attributed to its inherent physiological characteristics of high oxygen consumption and abundant unsaturated fatty acids. This renders it among the earliest involved and most severely damaged organs in the aging process [1,2]. Progressive cognitive decline is a hallmark feature of brain aging, with its core driving mechanism strongly linked to oxidative stress imbalance and sustained neuroinflammatory activation in brain tissue [3]. On one hand, the excessive accumulation of reactive oxygen species (ROS) in the brain during aging induces oxidative damage to lipids, proteins, and DNA. Concurrently, the declining functionality of endogenous antioxidant systems such as superoxide dismutase (SOD) and glutathione peroxidase (GSH-Px) further exacerbates this oxidative cascade, forming a vicious cycle [4]. On the other hand, the NF-κB signaling pathway remains persistently activated during brain aging, which drives the release of pro-inflammatory cytokines such as tumor necrosis factor-α (TNF-α) and interleukin-1β (IL-1β). This triggers neuroinflammation, accelerates neuronal apoptosis and synaptic dysfunction, and ultimately leads to cognitive impairment and other brain aging phenotypes [5,6].

The Nrf2/Keap1 pathway serves as the central antioxidant defense system in the brain. It helps maintain redox homeostasis and supports neuronal survival by regulating the expression of downstream antioxidant molecules, such as NAD(P)H quinone oxidoreductase 1 (NQO1) and heme oxygenase-1 (HO-1). Furthermore, the crosstalk between this pathway and the NF-κB inflammatory pathway directly modulates the balance of oxidative stress and neuroinflammation during brain aging. It thereby serves as a key molecular network regulating the progression of brain aging [7,8,9]. Thus, combined interventions targeting antioxidant and anti-inflammatory processes in brain are regarded as an effective strategy to delay brain aging and improve cognitive function.

With the increasingly widespread application of natural products in anti-aging research, cyanidin-3-O-glucoside (C3G), one of the most widely distributed and abundant core monomers in anthocyanins, exhibits promising potential for intervening in brain aging. This is attributed to its excellent antioxidant and anti-inflammatory activities as well as favorable blood–brain barrier penetration potential [10,11]. Previous studies have confirmed that anthocyanins and their major component C3G exert preventive effects on various neurological disorders, such as cerebral ischemia, Alzheimer’s disease, Parkinson’s disease, multiple sclerosis, and glioblastoma [11]. Furthermore, anthocyanins have been shown to ameliorate key pathological processes in aging, including neuronal apoptosis and necrosis, as well as learning and memory impairments [12,13,14]. While anthocyanins have been shown to regulate both the Nrf2 and NF-κB pathways [15,16,17], systematic evidence is still lacking for C3G. Specifically, its concurrent regulation of these pathways in the aging brain, the dose-dependency of these effects, and the integration of such molecular changes with improved behavioral outcomes remain to be fully established.

The D-galactose (D-gal)-induced aging mouse model has been widely used for the screening of anti-aging active substances and mechanism research, as it mimics key characteristics of natural aging, including enhanced oxidative stress, activated inflammatory responses, and organ dysfunction [18,19]. While this model cannot fully mimic the physiological evolutionary patterns of natural aging, it provides a reliable experimental tool for investigating the roles of oxidative stress and inflammation in aging-related cognitive decline. Based on this, the present study employed D-gal-induced aging mice as the model to systematically investigate the role of C3G in delaying brain aging. Specifically, behavioral tests were conducted to evaluate the protective effects of C3G on cognitive function. Additionally, histopathological analysis was performed to explore its impacts on hippocampal tissue. Furthermore, the levels of oxidative stress markers and inflammatory factors were determined to reveal the antioxidant and anti-inflammatory activities of C3G in the brain tissue. Finally, qRT-PCR and Western blot analyses were further used to elucidate the regulatory mechanisms of C3G on the Nrf2/Keap1 and NF-κB signaling pathways in hippocampal tissue. This study aims to systematically clarify the role and molecular mechanisms of C3G in delaying brain aging, thereby providing a theoretical foundation for its application in developing interventions against brain aging.

## 2. Materials and Methods

### 2.1. Materials

D-galactose (D-gal) was purchased from Beijing Biotopped Technology Co., Ltd. (Beijing, China). Cyanidin-3-O-glucoside (C3G) (purity = 94.59% as determined by HPLC) was obtained from Nanjing Jingzhu Biotechnology Co., Ltd. (Nanjing, China). Commercial kits for the determination of malondialdehyde (MDA), total antioxidant capacity (T-AOC), superoxide dismutase (SOD), Mn-SOD, reduced glutathione (GSH), and glutathione peroxidase (GSH-Px) in serum and brain tissue homogenates were all purchased from Nanjing Jiancheng Bioengineering Institute (Nanjing, China). Commercial kits for detecting inflammatory cytokines including interleukin-6 (IL-6), interleukin-1β (IL-1β), and tumor necrosis factor-α (TNF-α) in serum were acquired from MultiSciences Biotech Co., Ltd. (Hangzhou, China). Antibodies against Nrf2 (Cat No. 66504-1-Ig) and Keap1 (Cat No. 60027-1-Ig) were purchased from Proteintech Group, Inc. (Wuhan, China), while antibodies against IKKβ (Cat No. R1706-13) and NF-κB p65 (Cat No. ET1603-12) were obtained from Hangzhou Huabio Co., Ltd. (Hangzhou, China). All other chemical reagents used in this study were of analytical grade.

### 2.2. Animal Grouping and Intervention

Male C57BL/6J mice aged 6–8 weeks were purchased from Beijing Vital River Laboratory Animal Technology Co., Ltd. (Beijing, China) (Laboratory Animal Production License No.: SCXK (Jing) 2021-0006). All mice were housed under standard conditions (25 °C, 12 h light/dark cycle) with free access to food and water. All experimental protocols were approved by the Animal Experiment Ethics Committee of Beijing Technology and Business University (Approval No. Lunshen 2024 No. 163). After a 7-day acclimatization period, the mice were sorted by body weight and then allocated into different experimental groups using a snake-like randomization method to ensure comparable group mean weights. Ultimately, the mice were formally divided into four groups (*n* = 10 per group): normal control (NC) group, model group (500 mg/kg D-gal), low-dose C3G (C3G-L) intervention group, and high-dose C3G (C3G-H) intervention group. Mice in the NC group received a daily subcutaneous injection of normal saline (10 mL/kg), while those in the other groups received a daily subcutaneous injection of 5% (*w*/*v*) D-gal (500 mg/kg). Additionally, mice in the C3G-L and C3G-H groups were administered C3G via gavage at doses of 50 and 100 mg/kg, respectively. All treatments were performed once daily for 13 consecutive weeks.

### 2.3. Morris Water Maze (MWM) Assessment

The water maze experimental method referenced the research protocol by Qin et al. [20], employing the Morris Water Maze (MWM) test to evaluate spatial memory and long-term memory capabilities in mice. The maze (Model XR-XM101, Shanghai Xinruan Information Technology Co., Ltd., Shanghai, China) consisted of a circular pool (120 cm in diameter, 50 cm in height) filled with water, with a hidden platform (10 cm in diameter, 30 cm in height) submerged 1–2 cm under the surface. Experimental parameters were set as follows: 60 s swimming duration, 15 s platform residence time, “mouse experiment mode” selected, and the red boundary box adjusted to accurately frame the pool and platform. Distinct black-and-white markers of different shapes were placed on the four walls of the pool (North, South, East, West) to provide visual cues for spatial navigation. Notably, rodents achieve learning saturation with minimal individual variability on day 5 of consecutive training [21]. Accordingly, the MWM test was initiated at week 12 post-treatment and lasted 6 days, with days 1–5 devoted to acquisition training and day 6 to the spatial probe test. Acquisition training was conducted 1 h after daily gavage administration, with 4 training trials per mouse per day. Blinding was adopted throughout the experiment, and the personnel responsible for behavioral testing and data recording were unaware of the animal grouping information. For each trial, the mouse was placed into the water facing the pool wall from a predetermined quadrant, and the time taken to locate and mount the hidden platform (escape latency) was recorded. If a mouse found and remained on the platform for 15 s within the 60 s trial, its actual latency was recorded. Otherwise, the escape latency was recorded as 60 s, and the mouse was guided to the platform and allowed to remain there for 15 s to reinforce spatial memory. The interval between consecutive trials was 30–60 s. On day 6, the hidden platform was removed for the spatial probe test, which serves as an independent assessment of memory consolidation ability. Each mouse was released into the quadrant opposite the original platform location and allowed to swim freely for 60 s. The crossing frequency over the former platform location and the time spent exploring the target quadrant were recorded.

### 2.4. Sample Collection and Preparation

After the intervention period, mice were fasted for 12 h and then anesthetized via isoflurane inhalation. Following blood collection, mice were euthanized by cervical dislocation, and brain tissues were immediately dissected. Blood samples were centrifuged at 3500 rpm and 4 °C, for 15 min to separate serum, which was stored at −20 °C, for subsequent detection of inflammatory cytokines and antioxidant indices. The isolated brain tissues were rinsed with pre-cooled normal saline (0.9%, *w*/*v*), and surface moisture was blotted dry with sterile filter paper before being divided into three parts. The hippocampal region was rapidly dissected. One was fixed in 10% neutral buffered formalin for histopathological analysis, and the other was wrapped in aluminum foil and stored at −80 °C for subsequent qRT-PCR and Western blot analyses. The remaining brain tissue was homogenized in ice-cold saline (1:9, *w*/*v*) to prepare a 10% (*w*/*v*) tissue homogenate. The homogenate was centrifuged at 5000× *g* for 10 min at 4 °C, and the supernatant was collected and stored at −80 °C for antioxidant index determination.

### 2.5. Histopathological Evaluation of Hippocampal Tissue

Hippocampal tissue was fixed in 10% neutral buffered formalin solution for 24 h, followed by sequential tissue dehydration, xylene clearing, and paraffin embedding. Serial sections (5 μm thick) were cut and stained with Hematoxylin–eosin (H&E). Pathomorphological changes in the hippocampal tissue were observed and analyzed using a PANNORAMIC DESK/MIDI/250/1000 whole-slide scanner (3DHISTECH Ltd., Budapest, Hungary). The operators and analysts were unaware of the grouping information of the experimental animals to eliminate subjective bias in the evaluation process.

### 2.6. Determination of Antioxidant Indices

Quantitative determination of malondialdehyde (MDA), total antioxidant capacity (T-AOC), superoxide dismutase (SOD), manganese superoxide dismutase (Mn-SOD), reduced glutathione (GSH), and glutathione peroxidase (GSH-Px) levels was performed in serum and brain tissue homogenates. All aforementioned indices were measured using corresponding commercial kits, strictly following the manufacturers’ instructions. For brain tissue homogenate samples, all indices were calibrated based on the content per milligram of protein to standardize the results.

### 2.7. Detection of Inflammatory Cytokines

The serum concentrations of interleukin-6 (IL-6), interleukin-1β (IL-1β), and tumor necrosis factor-α (TNF-α) were quantified using mouse-specific sandwich enzyme-linked immunosorbent assay (ELISA) kits, strictly following the manufacturer’s protocols.

### 2.8. Quantitative Real Time PCR (qRT-PCR) Analysis

Total RNA was extracted from hippocampal tissue using the High-Purity Total RNA Rapid Extraction Kit (Tiangen Biotech, Beijing, China), strictly following the manufacturer’s instructions. All RNA samples were quantified using a Thermo Scientific NanoDrop^TM^ 2000 spectrophotometer (Thermo Fisher Scientific Inc., Waltham, MA, USA), and RNA integrity was verified via 1.5% agarose gel electrophoresis. Subsequently, 1 μg of the extracted RNA was reverse-transcribed into cDNA using the ReverTra Ace^®^ qPCR RT Master Mix (Toyobo Co. Ltd., Life Science Department, Osaka, Japan). qRT-PCR amplification and detection were conducted in Low-Profile PCR tubes using a Bio-Rad CFX96 Real-Time PCR System (Bio-Rad Laboratories, Inc., Hercules, CA, USA). Each 20 μL reaction system contained: 10 μL of SYBR^®^ Green Real-Time PCR Master Mix (Toyobo Co. Ltd., Life Science Department, Osaka, Japan), 10 μM of each forward and reverse primer, 1 μL of cDNA, and PCR-grade water to bring the volume to 20 μL. The thermal cycling protocol was set as follows: an initial polymerase activation step at 95 °C for 3 min, followed by 40 cycles of denaturation (94 °C for 30 s), annealing (60 °C for 40 s), and extension (72 °C for 1 min). Fluorescence was measured at the end of each annealing step to monitor amplification in real time. The specificity of the PCR products was confirmed by analyzing dissociation curves. Gene expression levels were normalized to the reference gene glyceraldehyde-3-phosphate dehydrogenase (GAPDH). Relative mRNA expression was calculated using the 2^−ΔΔCt^ method with 6 independent biological replicates performed for each experimental group. The primer sequences are provided in Table 1. The amplification efficiency for each primer pair was determined using a standard curve and was confirmed to be between 90% and 110%. The specificity of amplification was verified by sequencing the PCR products, which matched the expected target sequences in GenBank.

### 2.9. Western Blot Analysis

Approximately 20 mg of hippocampal tissue was homogenized thoroughly on ice in RIPA lysis buffer containing 1% protease inhibitor. After homogenization, the mixture was centrifuged at 4 °C, and the supernatant was collected. Protein concentration was determined using the BCA assay. Samples were mixed with 4× loading buffer at a 3:1 ratio, boiled for 5 min, and then cooled to room temperature for immediate use or stored at −80 °C. Proteins were separated by SDS-PAGE using a 10% separating gel and a 5% stacking gel, followed by transfer onto a PVDF membrane. The membrane was blocked with 5% non-fat milk at room temperature for 1 h and then incubated overnight at 4 °C with the following primary antibodies: Nrf2 (1:2000), Keap1 (1:2000), IKKβ (1:1000), NF-κB p65 (1:1000), and rabbit anti-β-actin (1:50,000). Subsequently, the membranes were incubated with species-appropriate HRP-conjugated secondary antibodies at a dilution of 1:20,000 for 1 h at room temperature. Protein bands were visualized using ECL chemiluminescent substrate and detected with a chemiluminescence imaging system. β-actin was used as the internal reference, and protein expression levels were normalized to the band intensity of β-actin. Quantitative analysis of band optical density was performed using ImageJ software (version 1.8.0).

### 2.10. Animal Inclusion/Exclusion Criteria and Sample Selection

The inclusion criteria required animals to be in good health, well-acclimated to the environment, and in a stable condition, with 10 animals initially allocated per group. Animals were excluded if they died unexpectedly during model establishment or exhibited significant abnormal behavioral responses during testing (e.g., frequent floating, immobility against the wall, or motor impairments). Consequently, behavioral and certain biochemical analyses were performed with *n* = 8 per group. For qRT-PCR, sample selection was based on predefined RNA quality control criteria. Only samples with A_260_/A_280_ ratios between 1.8–2.1, A_260_/A_230_ ratios > 2.0, and no evident degradation upon agarose gel electrophoresis were included, resulting in *n* = 6 per group for this analysis. For Western blot, due to the constraints of gel electrophoresis lanes and sample processing capacity, a stratified random sampling method was employed to select *n* = 3 representative samples per group. All sample exclusions and selections were conducted based on objective, pre-established criteria, independently of subsequent experimental outcomes, thereby avoiding selection bias. Sample sizes for each analysis are clearly indicated in the corresponding figure legends.

### 2.11. Statistical Analysis

All data are presented as mean ± standard deviation (SD). Normality and homogeneity of variance were confirmed via the Shapiro–Wilk and Levene’s tests, respectively, prior to parametric analyses. Specifically, repeated-measures ANOVA with Bonferroni post hoc multiple comparisons was used to assess time-course data (escape latency and swimming speed) during Morris water maze acquisition training. One-way ANOVA was performed, followed by Dunnett’s test to compare Western blot band gray values, while Duncan’s multiple range test was used for all other datasets. All statistical analyses were performed using SPSS 27.0, with statistical significance defined as *p* < 0.05.

## 3. Results

### 3.1. Protective Effects of C3G on Cognitive Function in D-Gal-Induced Aging Mice

**Figure 1:**
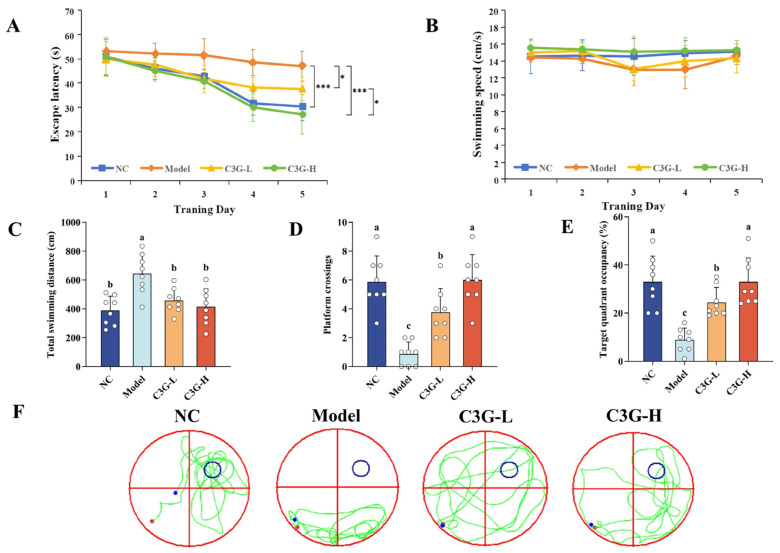
Effects of C3G on Morris water maze (MWM) performance in D-galactose (D-gal)-induced aging mice. (**A**) Escape latency, (**B**) swimming speed, and (**C**) total swim distance on the 5th day during 5 days of training trial. (**D**) Number of platform crossings, (**E**) percentage of time spent in the target quadrant, and (**F**) representative swimming trajectories in the spatial probe test. NC: Normal control group; Model: model group (500 mg/kg D-galactose); C3G-L: low-dose C3G intervention group (50 mg/kg); C3G-H: high-dose C3G intervention group (100 mg/kg). The data are presented as the mean ± SD, with *n* = 8 biological replicates per group. Time-course data were analyzed using repeated-measures ANOVA with Bonferroni post hoc tests (* *p* < 0.05, *** *p* < 0.001). Other data were assessed by one-way ANOVA followed by Duncan’s multiple range test. Different letters indicate significant differences (*p* < 0.05), while groups sharing the same letter or partial letters show no significant difference (*p* > 0.05).

The Morris water maze was used to evaluate the effects of C3G on spatial learning and long-term memory in D-gal-induced aging mice. As shown in Figure 1A, escape latency decreased across training days in all groups, indicating progressive learning. After five days of acquisition training, the escape latency of mice in the model group on the 5th day was longer (47.07 ± 6.23 s) compared with the NC group (30.41 ± 5.79 s, *p* < 0.001). The total swimming distance was also increased in the model group (637.42 ± 162.67 cm) relative to the NC group (385.15 ± 117.98 cm, *p* < 0.05) (Figure 1C). These results indicated that subcutaneous injection of D-gal accelerated aging and significantly impaired learning and memory abilities in mice, confirming the successful establishment of the aging model. Importantly, no significant difference in swimming speed was observed among the groups across all training days (*p* > 0.05), ruling out the influence of motor function on the results (Figure 1B). Compared with the model group, C3G intervention significantly shortened the escape latency on the 5th day, with the C3G-H group exhibiting the most prominent effect (*p* < 0.001). Its escape latency was reduced to 27.24 ± 8.93 s, representing a 42.07% reduction and comparable to the NC group. The total distance to reach the platform was also reduced in the C3G-H group (415.35 ± 125.27 cm, *p* < 0.05).

Results of the spatial probe test (Figure 1D,E) revealed that compared with the NC group, the model group exhibited a decrease in both the percentage of time spent in the target quadrant and the number of platform crossings (*p* < 0.05). In contrast, C3G intervention increased these two indices (*p* < 0.05), and the values in the C3G-H group were fully restored to the levels of the NC group (*p* > 0.05). Consistent with the acquisition training results, the spatial probe test further demonstrated that C3G exerted an protective effect on memory function in mice, with a more pronounced effect at the high dose. Representative swimming trajectory plots (Figure 1F) intuitively reflected the behavioral differences among groups. Mice in the model group mainly displayed thigmotactic and aimless swimming, while those in the C3G-H group exhibited an ordered search strategy centered on the target quadrant, similar to that of the NC group. Collectively, these findings confirmed that C3G significantly alleviates spatial memory impairment in D-gal-induced aging mice.

### 3.2. Effects of C3G on Hippocampal Tissue in D-Gal-Induced Aging Mice

**Figure 2:**
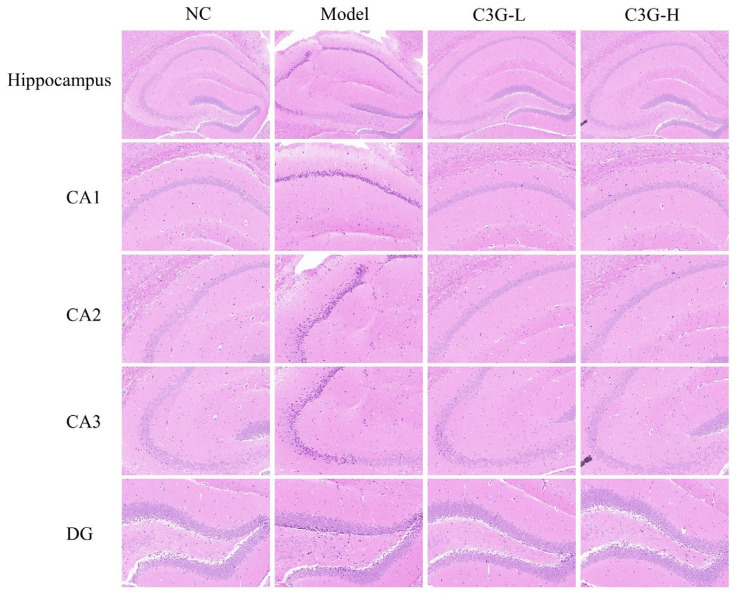
Effects of C3G on hippocampal histopathology in D-galactose (D-gal)-induced aging mice (HE staining, 200× magnification). NC: Normal control group; Model: model group (500 mg/kg D-galactose); C3G-L: low-dose C3G intervention group (50 mg/kg); C3G-H: high-dose C3G intervention group (100 mg/kg).

Hematoxylin–eosin (H&E) staining results of hippocampal tissue from each group are shown in Figure 2. In the NC group, neurons in the hippocampal *Cornu Ammonis* (CA) subregions CA1, CA2, CA3, and *Dentate Gyrus* (DG) subregions were neatly and densely arranged, with clear cell layer structure, plump cell morphology, distinct nuclear structure, and uniform chromatin distribution. No obvious pathological damage was observed. In contrast, representative sections of the model group exhibited sparse and disorganized arrangement of cell layers in the hippocampal CA1 and CA3 subregions, with increased intercellular spacing. Surviving neurons showed prominent apoptotic or necrotic features such as cellular shrinkage, pyknosis, and hyperchromatism. The granular cell layer of the DG subregion also displayed decreased cell density and disorganized arrangement. After C3G-L intervention, the pathological state of hippocampal tissue noticeable improvement. Neuronal arrangement exhibited a more orderly appearance. Although sporadic pyknosis remained observable, its occurrence was notably less frequent and severe compared to the model group, suggesting a potential neuroprotective effect of low-dose C3G. Notably, the improvement in pathological features was more pronounced in the C3G-H group. In representative sections of this group, the cellular morphology and structure of all hippocampal subregions (CA1, CA3, DG) were close to the NC group, with neurons arranged densely and intact in morphology. Pathological changes such as pyknosis were extremely rare. These observations from representative H&E-stained sections demonstrate that C3G treatment may alleviate hippocampal neuronal damage and improve the morphological integrity of neurons and tissue structure in D-gal-induced aging mice.

### 3.3. Protective Effects of C3G on Oxidative Stress in D-Gal-Induced Aging Mice

**Figure 3:**
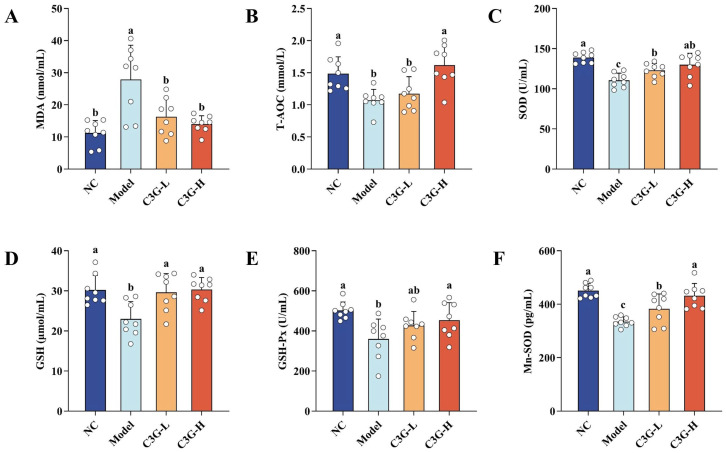
Effects of C3G on oxidative stress levels in serum of D-galactose (D-gal)-induced aging mice. (**A**) MDA, (**B**) T-AOC, (**C**) SOD, (**D**) GSH, (**E**) GSH-Px, and (**F**) Mn-SOD. NC: Normal control group; Model: model group (500 mg/kg D-galactose); C3G-L: low-dose C3G intervention group (50 mg/kg); C3G-H: high-dose C3G intervention group (100 mg/kg). The data are presented as the mean ± SD, with *n* = 8 biological replicates per group. Different letters indicated significant differences (*p* < 0.05), while groups sharing the same letter or partial letters show no significant difference (*p* > 0.05), as determined by one-way ANOVA followed by Duncan’s multiple range test.

To investigate the impact of C3G on oxidative stress status in aging mice, key antioxidant enzyme activities and lipid peroxidation levels were measured in serum and non-hippocampal brain tissue. As shown in Figure 3, compared with the NC group, the model group exhibited decreased activities of T-AOC, GSH, SOD, Mn-SOD and GSH-Px in serum (*p* < 0.05), while the MDA content was significantly increased (*p* < 0.05). These results indicate that the endogenous antioxidant capacity of mice in the model group was impaired, lipid peroxidation damage was exacerbated, and the aging model was successfully established. After intervention with different doses of C3G, both C3G-L and C3G-H groups showed significantly increased activities of SOD, GSH, and Mn-SOD compared with the model group (*p* < 0.05). In addition to these indices, the C3G-H group also exhibited elevated T-AOC and GSH-Px activities (*p* < 0.05), with all detected indicators restored to the levels of the NC group (*p* > 0.05). Meanwhile, the MDA content was reduced in both C3G-treated groups (*p* < 0.05). These findings demonstrate that the protective effect of C3G on oxidative stress in aging mice is dose-dependent, with the C3G-H group showing the most prominent regulatory effect, fully restoring antioxidant-related indices to normal levels.

**Figure 4:**
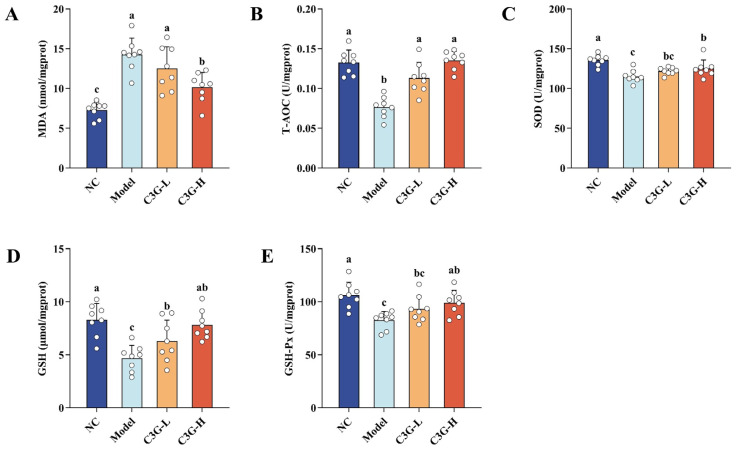
Effects of C3G on oxidative stress levels in non-hippocampal brain tissue of D-galactose (D-gal)-induced aging mice. (**A**) MDA, (**B**) T-AOC, (**C**) SOD, (**D**) GSH, and (**E**) GSH-Px. NC: Normal control group; Model: model group (500 mg/kg D-galactose); C3G-L: low-dose C3G intervention group (50 mg/kg); C3G-H: high-dose C3G intervention group (100 mg/kg). The data are presented as the mean ± SD, with *n* = 8 biological replicates per group. Different letters indicated significant differences (*p* < 0.05), while groups sharing the same letter or partial letters show no significant difference (*p* > 0.05), as determined by one-way ANOVA followed by Duncan’s multiple range test.

As an organ with high oxygen consumption and high lipid content, the brain is highly sensitive to oxidative stress and serves as a key target region for age-related cognitive decline. Non-hippocampal brain tissues were used to determine the following oxidative stress parameters. Compared with the NC group, the model group showed distinct oxidative brain damage (Figure 4), as indicated by an increase in MDA content and simultaneous reductions in T-AOC, GSH, SOD, and GSH-Px activities (*p* < 0.05). C3G intervention alleviated the abnormal oxidative stress in the model group. The C3G-L group exhibited higher T-AOC and lower MDA content (*p* < 0.05). Meanwhile, the C3G-H group showed reduced MDA content and elevated T-AOC, GSH, SOD, and GSH-Px activities (*p* < 0.05). More importantly, all its oxidative stress indices were restored to NC group levels (*p* > 0.05). These results further illustrate that C3G can effectively improve redox imbalance, inhibit lipid peroxidation, and thereby provide a mechanistic basis for its neuroprotective effect in ameliorating cognitive function in aging mice.

### 3.4. Effects of C3G on Serum Inflammatory Cytokine Levels in D-Gal-Induced Aging Mice

**Figure 5:**
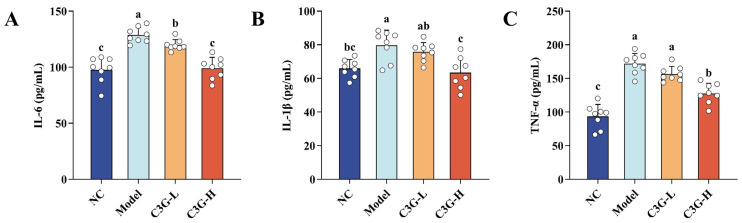
Effects of C3G on inflammatory cytokine levels in serum of D-galactose (D-gal)-induced aging mice. (**A**) IL-6, (**B**) IL-1β, and (**C**) TNF-α. NC: Normal control group; Model: model group (500 mg/kg D-galactose); C3G-L: low-dose C3G intervention group (50 mg/kg); C3G-H: high-dose C3G intervention group (100 mg/kg). The data are presented as the mean ± SD, with *n* = 8 biological replicates per group. Different letters indicated significant differences (*p* < 0.05), while groups sharing the same letter or partial letters show no significant difference (*p* > 0.05), as determined by one-way ANOVA followed by Duncan’s multiple range test.

As shown in Figure 5, the serum levels of key pro-inflammatory cytokines (TNF-α, IL-1β, and IL-6) were measured in each group of mice. Compared with the NC group, the model group exhibited increased levels of all tested cytokines (*p* < 0.05). This confirms that the D-gal-induced aging model successfully induced a systemic inflammatory state in mice. C3G intervention significantly reduced serum pro-inflammatory cytokine levels in aging mice in a dose-dependent manner. Specifically, while the C3G-H group showed suppression of TNF-α, IL-1β, and IL-6 levels (*p* < 0.05), only IL-6 was reduced in the C3G-L group (*p* < 0.05). These results demonstrate that C3G can effectively lower serum pro-inflammatory cytokine levels and inhibit systemic inflammatory responses in D-gal-induced aging mice. This may be one of the key mechanisms underlying its protective effects on age-related phenotypes in mice.

### 3.5. Regulatory Effects of C3G on mRNA Expression of Key Genes in the Nrf2/Keap1 Pathway in Hippocampal Tissue of D-Gal-Induced Aging Mice

**Figure 6:**
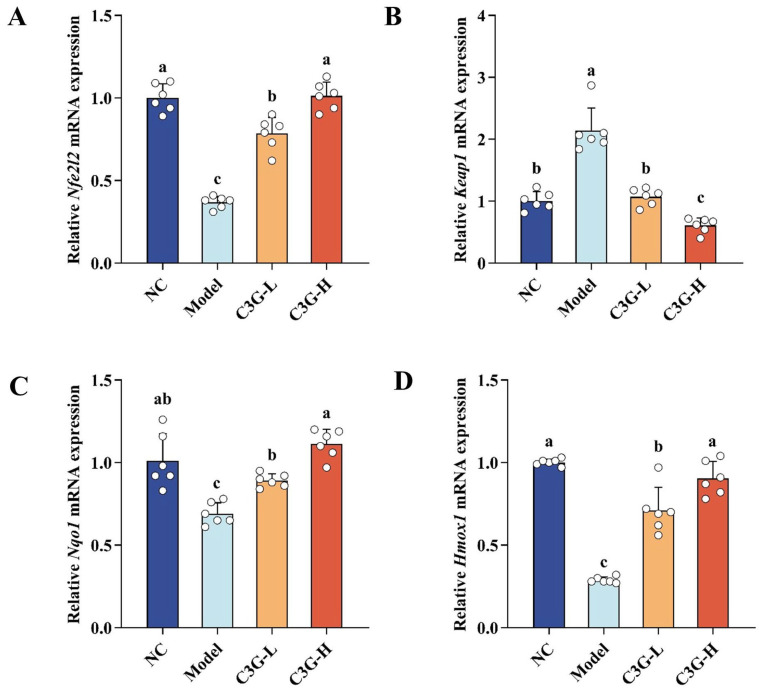
Effects of C3G on mRNA expression of key genes in the Nrf2/Keap1 pathway in hippocampal tissue of D-galactose (D-gal)-induced aging mice. (**A**) *Nfe2l2*, (**B**) *Keap1*, (**C**) *Nqo1*, and (**D**) *Hmox1*. NC: Normal control group; Model: model group (500 mg/kg D-galactose); C3G-L: low-dose C3G intervention group (50 mg/kg); C3G-H: high-dose C3G intervention group (100 mg/kg). The data are presented as the mean ± SD, with *n* = 6 biological replicates per group. Different letters indicated significant differences (*p* < 0.05), while groups sharing the same letter or partial letters show no significant difference (*p* > 0.05), as determined by one-way ANOVA followed by Duncan’s multiple range test.

As shown in Figure 6, compared with the NC group, the mRNA expression of *Nfe2l2* (encoding nuclear factor E2-related factor 2, Nrf2) was downregulated in the hippocampal tissue of the model group (*p* < 0.05). Meanwhile, the mRNA expression of its negative regulatory gene *Keap1* (encoding Kelch-like ECH-associated protein 1, Keap1) was upregulated (*p* < 0.05). Concurrently, the mRNA expression of Nrf2 downstream target genes, including *Nqo1* (encoding the phase II detoxifying enzyme NAD(P)H: quinone oxidoreductase 1) and *Hmox1* (encoding the antioxidant protein heme oxygenase-1), was also downregulated (*p* < 0.05). These results indicate that D-gal-induced aging can significantly inhibit the transcriptional expression of the Nrf2/Keap1 antioxidant pathway in the hippocampal tissue of mice. C3G intervention effectively attenuated this transcriptional suppression in both the C3G-L and C3G-H groups. Specifically, *Keap1* mRNA expression was reduced (*p* < 0.05), while the mRNA expressions of *Nfe2l2* and its downstream target genes *Nqo1* and *Hmox1* were increased (*p* < 0.05). These findings demonstrate that C3G may enhance endogenous antioxidant defense in hippocampal tissue by potentially activating Nrf2 signaling at the transcriptional level, and subsequently upregulating the expression of downstream antioxidant-related target genes *Nqo1* and *Hmox1*.

### 3.6. Regulatory Effects of C3G on mRNA Expression of Key Genes in the NF-κB Pathway of Hippocampal Tissue in D-Gal-Induced Aging Mice

**Figure 7:**
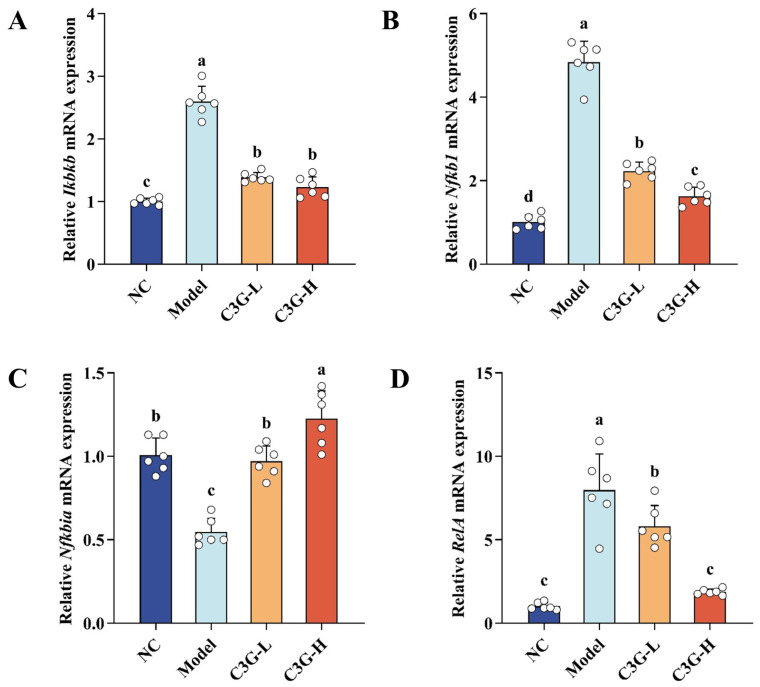
Effects of C3G on mRNA expression of key genes in the NF-κB pathway in hippocampal tissue of D-galactose (D-gal)-induced aging mice. (**A**) *Ikbkb*, (**B**) *Nfkb1*, (**C**) *Nfkbia*, and (**D**) *RelA*. NC: Normal control group; Model: model group (500 mg/kg D-galactose); C3G-L: low-dose C3G intervention group (50 mg/kg); C3G-H: high-dose C3G intervention group (100 mg/kg). The data are presented as the mean ± SD, with *n* = 6 biological replicates per group. Different letters indicated significant differences (*p* < 0.05), while groups sharing the same letter or partial letters show no significant difference (*p* > 0.05), as determined by one-way ANOVA followed by Duncan’s multiple range test.

As shown in Figure 7, compared with the NC group, the model group exhibited upregulated mRNA expression levels of NF-κB signaling pathway key genes *Ikbkb* (inhibitor of nuclear factor kappa-B kinase subunit beta, IKKβ), *Nfkb1* (nuclear factor kappa-B subunit 1, p50) and *RelA* (v-rel avian reticuloendotheliosis viral oncogene homolog A, p65) in the hippocampal tissue (*p* < 0.05). In contrast, the mRNA expression level of its inhibitory gene *Ikbα* (nuclear factor of kappa light polypeptide gene enhancer in B-cells inhibitor alpha) was downregulated (*p* < 0.05). These results indicate that the NF-κB inflammatory pathway exhibited marked transcriptional upregulation in the hippocampal tissue of D-gal-induced aging model mice, suggesting a state of chronic inflammation. After C3G intervention, the mRNA expression levels of *Ikbkb*, *Nfkb1* and *RelA* were all reduced compared with the model group (*p* < 0.05). This transcriptional regulatory effect showed a clear dose dependence. Notably, all aforementioned mRNA indicators in the C3G-H group were restored to levels that did not differ significantly from the NC group (*p* > 0.05). The findings suggest that C3G may attenuate the transcriptional upregulation of the NF-κB pathway in the hippocampal tissue of mice by potentially modulating the IKKβ/IκBα/p65 signaling axis at the transcriptional level.

### 3.7. Regulatory Effects of C3G on Key Protein Expression in the Nrf2 and NF-κB Pathways in Hippocampal Tissue of D-Gal-Induced Aging Mice

**Figure 8:**
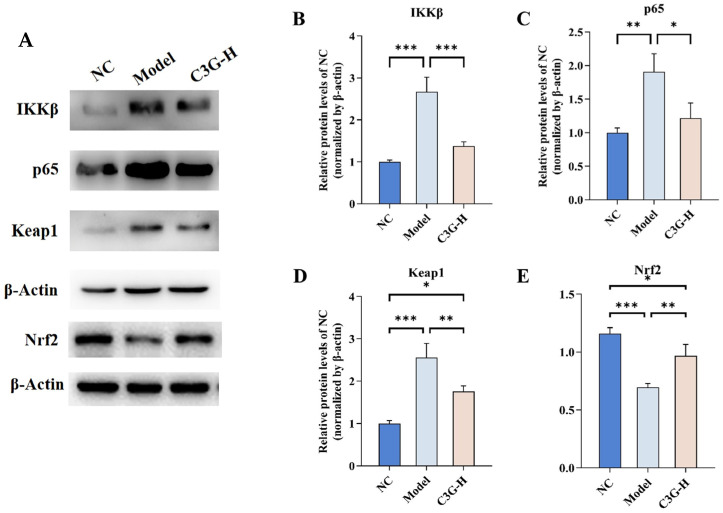
Effects of C3G on the expression of key proteins in the Nrf2 and NF-κB pathways in hippocampal tissue of D-galactose (D-gal)-induced aging mice. (**A**) Representative Western blot images. Relative expression levels of IKKβ (**B**), p65 (**C**), Keap1 (**D**) and Nrf2 (**E**) compared to NC (normalized by β-actin). NC: Normal control group; Model: model group (500 mg/kg D-galactose); C3G-H: high-dose C3G intervention group (100 mg/kg). The data are presented as the mean ± SD, with *n* = 3 biological replicates per group. Data were tested for normality using the Shapiro–Wilk test (*p* > 0.05). Statistical analysis was performed using one-way ANOVA. Dunnett’s test was used for post hoc comparisons, and significance levels are indicated as * *p* < 0.05, ** *p* < 0.01, and *** *p* < 0.001.

To further validate the regulatory effects of C3G on the Nrf2 and NF-κB pathways in the hippocampus of D-gal-induced aging mice, this study selected the high-dose C3G (C3G-H) intervention group for subsequent analysis, focusing on key targets of both pathways based on the qRT-PCR findings. As shown in Figure 8, compared with the NC group, the protein expression of Nrf2 was downregulated in the brain of the model group (*p* < 0.001). Meanwhile, the expressions of Keap1 (*p* < 0.001), IKKβ (*p* < 0.001) and the NF-κB transcription factor p65 (*p* < 0.01) were upregulated. These alterations collectively indicate a state of chronic inflammation associated with impaired antioxidative capacity in D-gal-induced aging mice. In the C3G-H group, protein levels of IKKβ (*p* < 0.001), p65 (*p* < 0.05) and Keap1 (*p* < 0.01) were all decreased, while Nrf2 protein expression (*p* < 0.01) was increased relative to the model group. Notably, the protein expression levels of IKKβ and p65 were even restored to those of the normal control group (*p* > 0.05). Collectively, these results support that C3G treatment was associated with enhanced endogenous antioxidant defenses in hippocampal tissue via changes consistent with inhibition of Keap1 and activation of Nrf2, while also reducing chronic inflammation through modulation of key proteins involved in the IKKβ/p65 signaling. These observations may provide a potential molecular mechanism underlying the neuroprotective and anti-brain aging effects of C3G.

## 4. Discussion

This study employed a D-gal-induced aging mouse model to systematically investigate the improving effects of C3G on aging-related cognitive impairment and its underlying molecular mechanisms. In this study, male mice were employed for model construction, with the aim of controlling confounding variability associated with sex differences and the estrous cycle of females in the stage of initial mechanistic inquiry. Collectively, our results demonstrate that C3G dose-dependently improved D-gal-induced cognitive dysfunction and hippocampal histopathological damage. Importantly, these protective effects were linked to the concurrent modulation of two key pathways, specifically involving the activation of the Nrf2/Keap1 antioxidant pathway and the suppression of the NF-κB inflammatory pathway in the hippocampal tissue. The conclusions of this study are derived from a male accelerated aging model, which is artificially induced and characterized by rapid, targeted pathological changes that are distinct from the slow, systemic degeneration observed in natural aging. Since elevated oxidative stress and chronic inflammation are shared core mechanisms between accelerated and physiological aging, the ability of C3G to regulate these pathways suggests its potential as an intervention for mitigating cognitive decline during natural aging. However, it should be emphasized that the accelerated aging model cannot fully recapitulate the systemic, progressive hallmarks of natural aging. Future studies are needed to validate the effects of C3G on inflammation and cognition in female animals, and more crucially, in natural aging models, to comprehensively assess the generalizability of its potential applications.

Decline in spatial learning and memory is a hallmark phenotype of aging-related cognitive impairment. As the core brain region mediating memory encoding and retrieval, the structural integrity and functional activity of the hippocampal CA1, CA3 and DG subregions directly determine the body’s spatial cognitive ability [22]. In this study, D-gal treatment prolonged the escape latency of mice in the Morris water maze, reduced the percentage of time spent in the target quadrant and the number of crossings over the former platform location. Concurrently, histopathological examination revealed nuclear pyknosis, disordered arrangement, and blurred nuclear-cytoplasmic boundaries in the hippocampal CA1, CA3, and DG regions. These observations are consistent with the established model characteristics of D-gal-induced brain aging and cognitive impairment, which involve oxidative stress, neuroinflammation, and neuronal damage [23]. C3G intervention significantly alleviated the aforementioned behavioral and histological abnormalities. The high-dose intervention was particularly effective, not only elevating cognitive function to near-normal levels but also largely preserving the structure and number of hippocampal neurons. This finding aligns with previous studies demonstrating the protective effects of anthocyanins on cognitive function in animal models of aging and neurodegenerative diseases [12,24,25]. These results suggest that C3G can effectively alleviate D-gal-induced age-related cognitive impairment in mice by mitigating hippocampal neuronal damage. This effect may be attributed to the ability of C3G to cross the blood–brain barrier (BBB) and accumulate in brain tissue [26]. However, the present study did not perform quantitative analysis of the actual exposure levels of C3G in the brain. To further clarify C3G’s neuroprotective role and underlying mechanisms, future studies should include systematic pharmacokinetic evaluations, as well as in vivo electrophysiology and assessments of synaptic plasticity markers in aging models.

Oxidative stress imbalance is one of the core mechanisms underlying D-gal-induced aging. Owing to its high oxygen demand and substantial lipid content, the brain is a susceptible target organ for oxidative damage [27]. Our study found that D-gal treatment significantly inhibited the activities of antioxidant enzymes such as SOD and GSH-Px in the serum and non-hippocampal brain tissue of mice, and increased the level of malondialdehyde (MDA), a lipid peroxidation product. Meanwhile, D-gal treatment was associated with downregulated mRNA and protein expression of Nrf2, as well as mRNA expression of its downstream target genes *Nqo1* and *Hmox1*, whereas it upregulated mRNA and protein levels of the negative regulator Keap1 in the hippocampal tissue. In contrast, C3G treatment elevated antioxidant enzyme activities and reduced MDA content in a dose-dependent manner. In the C3G-H group, it was found to suppress Keap1 expression and increase Nrf2 expression, findings that are consistent with Nrf2 pathway activation. These observations support the ability of C3G to enhance the endogenous antioxidant defense system. These results are consistent with the findings reported by Chen et al. [28], who confirmed that anthocyanins can alleviate oxidative stress in the organism by activating the Nrf2/Keap1 pathway. The alterations suggest that the Nrf2/Keap1 pathway may contribute to the protective effects of C3G against oxidative damage and neuronal injury. Furthermore, our previous study demonstrated that C3G could bind to key amino acid residues (e.g., Arg415, Ser602) of Keap1, thereby disrupting Keap1-Nrf2 interaction and stabilizing Nrf2 for nuclear translocation [29]. Notably, the induced expression of Nrf2 downstream target gene *Hmox1* is crucial. HO-1 exhibits direct antioxidant activity, and its catalytic products (e.g., carbon monoxide and biliverdin) also possess anti-inflammatory and anti-apoptotic properties [30]. This feature further links antioxidant activity closely with anti-inflammatory and neuroprotective effects. Notably, as behavioral assessments focused on hippocampus-dependent functions, hippocampal tissues were prioritized for histopathological and molecular analyses to link molecular changes to behavioral phenotypes. Due to limited tissue availability, non-hippocampal samples (including cortex) were used for oxidative stress marker detection. Although the cortex contributes to cognition, its biochemical measures do not fully align with behavioral outcomes. Future studies should validate oxidative changes directly in the hippocampus and clarify its interplay with the cortex.

Chronic neuroinflammation is a key driver of aging-related cognitive impairment. Aberrant activation of the NF-κB pathway represents the core mechanism underlying neuroinflammation [31]. In the present study, we observed that D-gal-induced aging in mice was associated with upregulated mRNA expression of key NF-κB pathway-related genes (*Ikbkb*, *Nfkb1*, *RelA*) and increased protein levels of their encoded products IKKβ and p65, as well as downregulated mRNA expression of Ikbα in hippocampal tissue. Meanwhile, serum levels of pro-inflammatory cytokines such as TNF-α, IL-1β, and IL-6 were significantly elevated. These observations are consistent with a state of concurrent systemic inflammation and neuroinflammation in D-gal-induced aging mice. Notably, C3G dose-dependently downregulated mRNA expression of the key genes (*Ikbkb*, *Nfkb1*, *RelA*) in hippocampal tissue and suppressed the release of pro-inflammatory cytokines. Moreover, C3G administration at the high dose significantly decreased protein levels of IKKβ and p65. Together, these observations contributed to the alleviation of neuroinflammation. These findings are consistent with previous studies reporting that C3G and other anthocyanins may alleviate inflammation by regulating the NF-κB pathway, as reflected by decreased levels of IL-6 and TNF-α in models of neurodegenerative disease and aging-related inflammation [11,32,33]. Therefore, the downregulation of the upstream kinase IKKβ and the downstream p65 by C3G observed in this study is associated with attenuated inflammatory signaling in this pathway. Additionally, this dose-dependent regulatory pattern is consistent with the principle proposed by that natural bioactive components exert local regulation at low doses and comprehensive intervention on inflammatory pathways at high doses [34]. It further supports the specificity and efficacy of C3G’s anti-inflammatory effects.

**Figure 9:**
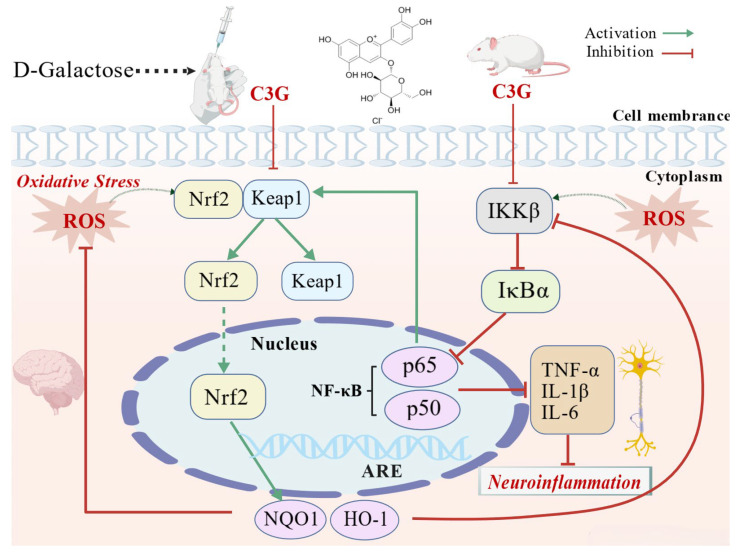
A working model illustrating the potential protective mechanism of cyanidin-3-O-glucoside (C3G) in counteracting cognitive impairment in D-galactose-induced aging mice by regulating Nrf2 and NF-κB pathways.

This study supports a potential working model by which C3G may counteract brain aging through the integration of Nrf2/NF-κB pathway regulation, along with hippocampal structural protection, and behavioral function recovery (Figure 9). On one hand, Nrf2-related expression changes elevate antioxidant levels such as GSH and establish a reductive cellular microenvironment. By scavenging ROS, these changes indirectly inhibit IKKβ activity and block the ROS–inflammation vicious cycle [35,36]. Meanwhile, previous studies have indicated that Nrf2 directly competes with p65 for the essential transcriptional coactivators CBP/p300, thereby physically inhibiting NF-κB’s transcriptional activity [37,38]. These dual effects collectively alleviate oxidative stress and inflammatory damage, reduce hippocampal neuronal and synaptic damage, and thereby preserve the structural integrity of hippocampal tissue. On the other hand, C3G downregulates NF-κB pathway-related components, which may in turn reduce pro-inflammatory cytokine release and suppress NADPH oxidase activation, thereby mitigating the oxidative burst associated with excessive ROS production during inflammation [35]. Such regulation may help preserve hippocampal structural stability and create a favorable cellular microenvironment for sustained antioxidant defense. These effects may, in turn, contribute to the behavioral improvements in cognitive function. These findings expand our understanding of C3G’s nutritional functions and offer new insights into dietary strategies against aging-related cognitive impairment. Future studies examining nuclear translocation of these key proteins and the crosstalk between Nrf2 and NF-κB pathways will help clarify the underlying mechanisms in the aging brain.

## 5. Conclusions

This study demonstrates that C3G dose-dependently alleviates cognitive impairment and hippocampal pathological damage in D-gal-induced aging mice. The potential mechanism is associated with the modulation of oxidative stress and inflammatory responses, as evidenced by activation markers of the Nrf2/Keap1 pathway and suppression markers of the NF-κB pathway. These two pathways appear to act together in improving cognitive impairment. By scavenging ROS, a known NF-κB activator, the Nrf2-mediated antioxidant response may suppress NF-κB markers and reduce ROS burst, thereby breaking the vicious cycle and alleviating cognitive deficits. In conclusion, C3G exhibits significant potential in delaying D-gal-induced brain aging and related cognitive decline. Future studies should clarify the crosstalk mechanism between the Nrf2 and NF-κB pathways under C3G intervention. They should also use techniques such as in vivo electrophysiology to directly evaluate C3G’s effects on hippocampal synaptic plasticity. This will provide crucial theoretical support for developing C3G-based nutritional intervention strategies.

## Figures and Tables

**Table 1:** Sequences of real-time PCR primers.

Gene	Species	Sequence (5′ --> 3′)	Amplicon Size (bp)
*Nfe2l2*	Mouse	F: GGACTACAGTCCCAGCAGAGTG R: CGGAAGGTTACAACGTGGGG	188
*Keap1*	Mouse	F: ATGTTGACACGGAGGATTGG R: TCATCCGCCACTCATTCCT	133
*Nqo1*	Mouse	F: GAAGACATCATTCAACTACGCC R: GAGATGACTCGGAAGGATACTG	179
*Hmox1*	Mouse	F: TCCTTGTACCATATCTACACGG R: GAGACGCTTTACATAGTGCTGT	198
*Ikbkb*	Mouse	F: GCAGACTGACATTGTGGACCTG R: ATCTCCTGGCTGTCACCTTCTG	150
*Nfkbia*	Mouse	F: ATCCTGACCTGGTTTCGCTC R: CTGTATCCGGGTACTTGGGC	113
*Nfkb1*	Mouse	F: ATGGCAGACGATGATCCCTAC R: TGTTGACAGTGGTATTTCTGGTG	111
*RelA*	Mouse	F: TCTGCCGAGTAAACCGGAAC R: AGCCTGGTCCCGTGAAATAC	111
*Gapdh*	Mouse	F: TCAACGGCACAGTCAAGG R: ACTCCACGACATACTCAG	126

## Data Availability

The original contributions presented in this study are included in the article. Further inquiries can be directed to the corresponding author.
